# Supplementary material for: Comparison of visceral adipose tissue DNA methylation and gene expression profiles in female adolescents with obesity
Source: Diabetol Metab Syndr. 2019 Nov 27;11:98. doi: 10.1186/s13098-019-0494-y (PMC6881970; doi:10.1186/s13098-019-0494-y)
Supplement: Supplementary file 1 — Additional file 1: Table S1. PCR confirmation cohort clinical characteristics. [file 13098_2019_494_MOESM1_ESM.docx]

| Additional Table S1. PCR Confirmation Cohort. | | | | | |
| --- | --- | --- | --- | --- | --- |
|  | **n** | **Age** | **Height** | **Weight** | **BMI** |
| **Lean** | 15 | 15 ± 3 | 160 ± 8 | 55 ± 11 | 21.5 ± 2.7 |
| **African American** | 5 | 16 ± 3 | 162 ± 8 | 60 ± 14 | 22.5 ± 3.9 |
| **Caucasian** | 5 | 16 ± 2 | 162 ± 7 | 56 ± 11 | 21.1 ± 2.1 |
| **Hispanic** | 6 | 12 ± 2 | 156 ± 8 | 51 ± 10 | 21 ± 2.2 |
| **Obese** | 19 | 17 ± 2 | 163 ± 8 | 130 ± 29 | 48.4 ± 9.3 |
| **African American** | 6 | 17 ± 3 | 166 ± 6 | 144 ± 20 | 52.8 ± 9.2 |
| **Caucasian** | 7 | 16 ± 2 | 163 ± 10 | 112 ± 39 | 41.4 ± 9.1 |
| **Hispanic** | 6 | 17 ± 1 | 162 ± 8 | 130 ± 24 | 49.6 ± 7.7 |
